# American Medical Association

**Published:** 1893-05

**Authors:** 


### Society Meetings.

### AMERICAN MEDICAL ASSOCIATION.

### SECTION ON NEUROLOGY AND MEDICAL JURISPRUDENCE.

Preliminary programme of the annual meeting, to be held at Milwaukee, Wisconsin, June 6, 7, 8, and 9, 1893.

Officers of the Section : Charles K. Mills, M. D., 1,909 Chestnut street, Philadelphia, Pa., Chairman ; James G. Kiernan, M. D., 834 Opera House Block, Chicago, Illinois, Secretary. Executive Committee : O. Everts, M. D., Cincinnati, Ohio ; H. N. Moyer, M. D., Chicago, Illinois ; Justin E. Emerson, M. D., Detroit, Michigan.

The American Medical Association will meet at Milwaukee, Wisconsin, June 6, 7, 8, and 9, 1893. A preliminary programme of the Section of Neurology and Medical Jurisprudence has been prepared and is appended, and it will be seen that the meeting promises to be one of great interest. The first session will be held on the afternoon of June 6th. Two sessions will be held June 7th and 8th, one in the morning and one in the afternoon. The last session will be held on the morning of June 9th. Papers will be accepted for the final programme until May 1st, but not later, as all titles must be sent to the Chairman or Secretary by this date in order to allow sufficient time for the preparation of the programmes for the meeting of the entire Association. If you have not yet indicated your intention to take part, you are earnestly requested to contribute a paper, to present cases, or to exhibit gross or microscopical specimens. It is desired by many of the members of the section to have a dinner on one of the evenings of the meeting, the subscription to which will be $3.00. If you favor this proposition, please notify the Chairman or Secretary of your willingness to subscribe.

Dr. William Osler, Baltimore, Md., Anorexia Nervosa; Dr. Irving C. Rosse, Washington, D. C., Evidences of Paranoia Gleaned from the United States Patent Office; Dr. Harold N. Moyer, Chicago, Ill., Acromegaly; Dr. Henry H. Donaldson, Chicago, Ill., On the Weight of the Brain; Dr. Harriet C. B. Alexander, Chicago, Ill., Paretic Dementia in Women; Dr. Daniel R. Brower, Chicago, Ill., Suggestions on the Treatment of Sclerosis of the Spinal Cord; Dr. Archibald Church, Chicago, Ill., I.—Occupation Neuroses Affecting the Muscles of the Neck. II.—Syringomyelia. Dr. James G. Kiernan, Chicago, Ill., Malpractice in Insane Hospitals; Dr. L. Harrison Mettler, Chicago, Ill., I.—Hemiparaplegia; Report of a Case Completely Recovered after One Year's Duration. II.—Aural Vertigo (Meniere's Disease). Dr. E. S. Talbot, Chicago, Ill., Race Degeneracy and the Jaws; Dr. G. F. Lydston, Chicago, Ill., Remarks on the Therapeutical Use of Static Electricity; Dr. T. H. McBride, Milwaukee, Wis., Thoughts on the Causation of Insanity; Dr. James J. Putnam, Boston, Mass., Recent Discoveries and Observations Bearing on the Subject of Poisoning from Exposure to Arsenical Wall Papers; Dr. Thomas D. Crothers, Hartford, Conn., American Inebriate Asylums; Dr. E. D. Fisher, New York, N. Y., Transverse Myelitis; Dr. Landon Carter Gray, New York, N. Y., What Should Constitute Legal Responsibility, in the Medical Sense, in Insanity; Dr. Graeme M. Hammond, New York, N. Y., On the Proper Method of Determining Whether an Alleged Lunatic Shall be Declared Legally Insane or Not; Dr. Frederick Peterson, New York, N. Y., Care of Epileptics; Dr. Bernard Sachs, New York, N. Y., Syphilis of the Cord Simulating Tabes; Dr. Thomas G. Morton, Philadelphia, Pa., Some Medico-Legal Experiences in Railroad Cases; Dr. Wharton Sinkler, Philadelphia, Pa., Some Points in the Weir-Mitchell Rest Treatment; Dr. James Hendrie Lloyd, Philadelphia, Pa., A Study of Gliomatous Processes in the Spinal Cord (Illustrated by Microscopical Sections); Dr. Francis X. Dercum, Philadelphia, Pa., The Symptomatology of Cerebellar Tumor; Dr. Charles A. Oliver, Philadelphia, Pa., A Study of the Ocular Symptoms in Friedreich's Disease; Dr. Hobart A. Hare, Philadelphia, Pa., Has the So-called Suspension Treatment of Diseases of the Spinal Cord Proved an Addition to our Therapeutics? Dr. J. Madison Taylor, Philadelphia, Pa., I.—Notes on the Treatment of Exophthalmic Goitre. II.—Insanity in Childhood. Dr. Charles W. Burr, Philadelphia, Pa., A Contribution to the Study of Friedreich's Ataxia; Dr. D. D. Stewart,

Philadelphia, Pa., The Diagnosis of Lead Convulsions ; Dr. John B. Deaver, Philadelphia, Pa., A Consideration of the Different Trigeminal Operations for the Relief of Pain ; Dr. Henry Leffmann, Philadelphia, Pa., Experiences of a Chemist with Delusional Insanity ; Dr. Charles K. Mills and Dr. G. E. de Schweinitz, Philadelphia, Pa., Hemianopsia and Certain Symptom-Groups in Sub-Cortical Lesions; Dr. Charles K. Mills, Philadelphia, Pa., Paranoia in some of its Medico-Legal Aspects ; Dr. Isaac N. Kerlin, Elwyn, Pa., Early Recognition and Rational Treatment of Moral Imbecility ; Dr. Theodore Diller, Pittsburgh, Pa., A Case of Sub-Cortical Cyst of the Lower Part of the Ascending Parietal Convolution—Operation—Recovery ; Dr. Frank T. Norbury, Jacksonville, Ill., Insanity of the Aged ; Dr. Annette McFarland, Jacksonville, Ill., Gynecology in the Insane ; Dr. C. H. Hughes, St. Louis, Mo., Dyspepsia as a Nervous Disease : or, Indigestion in its Nervous Aspects and Relations ; Dr. J. T. Eskridge, Denver, Col., Case of Syphilis of the Pia, Simulating Tumor of the Brain ; Mono-Spasm and Mono-Paresis—Operation—Death on the Third Day. Dr. H. A. Tomlinson, St. Peter, Minn., The Inadequacy of the Morbid Anatomical Changes Found Post-Mortem to Explain the Manifestations of Insanity ; Dr. R. M. Phelps, Rochester, Minn., Degrees of Responsibility as Found in the Insane; Dr. C. B. Burr, Pontiac, Mich., Surgery in the Insane ; Dr. T. L. Wright, Bellefontaine, Ohio, The Special Influence of Alcohol on the Body.

